# Interchromosomal Translocations and Large Deletions Drive the Evolution of the Outlier Chromosome in the Smallest Photosynthetic Eukaryote

**DOI:** 10.1093/gbe/evag162

**Published:** 2026-07-02

**Authors:** Claire Bugnot, Tyler Alioto, Fernando Cruz, Eric Manirakiza, Jessica Gomez Garrido, Marta Gut, Sheree Yau, Gwenael Piganeau

**Affiliations:** Sorbonne University, CNRS, University Via Domitia, LBBM (UMR 8176), Banyuls sur Mer, France; Centre de Regulació Genòmica, Barcelona 08028, Spain; Centre de Regulació Genòmica, Barcelona 08028, Spain; Sorbonne University, CNRS, University Via Domitia, LBBM (UMR 8176), Banyuls sur Mer, France; Centre de Regulació Genòmica, Barcelona 08028, Spain; Centre de Regulació Genòmica, Barcelona 08028, Spain; Universitat de Barcelona (UB), Barcelona, Spain; Sorbonne University, CNRS, University Via Domitia, LBBM (UMR 8176), Banyuls sur Mer, France; Sorbonne University, CNRS, University Via Domitia, LBBM (UMR 8176), Banyuls sur Mer, France

**Keywords:** structural variants, chromoanagenesis, experimental evolution, phytoplankton, picoeukaryote, Nucleocytoviricota

## Abstract

Marine microalgal populations can rapidly evolve resistance to viruses upon infection. In *Ostreococcus mediterraneus*, resistance to the prasinovirus OmV2 emerged within 5 d in all virus-exposed populations. Whole-genome sequencing of pairs of resistant and susceptible cell lines revealed extensive structural genomic changes, particularly on the small outlier chromosome (SOC). SOC alterations included large deletions, duplications, rearrangements, and whole-chromosome duplication, yet no consistent structural variant or single nucleotide polymorphism was directly associated with resistance. Hybrid de novo assemblies confirmed the unique SOC assembly of each strain, with a highly polymorphic ∼2 kb tandem repeat region exhibiting an “accordion-like” pattern of expansion and contraction. No new viral insertions were found, though endogenous viral elements were conserved across lines. Two interchromosomal translocations between the SOC and chromosomes 2 and 17 offer novel insights into the mechanisms underlying the distinctive evolutionary path of this chromosome. Together, these findings demonstrate that resistance to OmV2 evolves rapidly and consistently but cannot yet be attributed to any specific structural variations, suggesting that transcriptional or posttranscriptional mechanisms underlie the resistant phenotype. Instead, the high rate of localized genomic structural variations points to a distinct mechanism of chromosome evolution.

Significance The mechanisms and consequences of structural variation remain poorly understood in many lineages, particularly in ecologically important marine microbes. This study shows that resistance to prasinovirus OmV2 in a cosmopolitan phytoplankton species, *Ostreococcus mediterraneus*, evolves rapidly and consistently, even though no resistance-associated single nucleotide polymorphisms or shared structural variants were identified on standard chromosomes. Instead, extensive genomic rearrangements—particularly of a single chromosome—occur independently of viral infection. These findings highlight the role of chromoplexy as the driving mechanism underlying the diversification of the enigmatic small outlier chromosome in this lineage.

## Introduction

Genetic diversity underlies environmental adaptation (Pellestor and Gatinois 2020). It arises from both microevolutionary processes—such as single nucleotide polymorphisms (SNPs) and small insertion–deletions (indels)—and macroevolutionary processes, also known as structural variations (Alkan et al. 2011). Structural variations are typically defined as genomic differences larger than 50 bp, encompassing insertions, deletions, duplications, inversions, translocations, and large tandem repeats (Alkan et al. 2011; Kosugi et al. 2023; Hu et al. 2025). Structural variations are induced by various molecular processes such as replication, repair mechanisms, and DNA recombination (Currall et al. 2013). In some cases, these events can result in extensively rearranged chromosomes. Initially identified as the underlying cause of numerous genetic diseases in humans (Stankiewicz and Lupski 2010; Lupski 2023), structural variations are now also recognized as an important source of genetic diversity (Stankiewicz and Lupski 2010; Kloosterman and Cuppen 2013; Yi and Ju 2018; Koltsova et al. 2019; Peng et al. 2019; Dobry et al. 2023; Bian et al. 2024; Nair et al. 2025).

The decreasing cost and increasing accessibility of long-read sequencing technologies have provided an unprecedented access to the study of structural variation. Large structural variation leading to significant karyotypic changes, such as changes in chromosome number and length, has been reported between isolated populations from the same species and may accumulate with time (Hron et al. 2026; Kellenberger et al. 2026; Totikov et al. 2026), and these large structural variations may ultimately lead to sub-species and speciation. In contrast to the gradual accumulation of structural variants between sub-population and species, the analysis of cell lines has revealed burst of structural rearrangements within few cell divisions. Catastrophic chromosomal rearrangements have been collectively referred to as chromoanagenesis, from Greek “chromo” meaning chromosome and “anagenesis” meaning rebirth (Holland and Cleveland 2012). Three types of structural rearrangements have been identified from cancer cells: chromothripsis (“thripsis” for shattering [Stephens et al. 2011]), chromoanasynthesis (“anagenesis” for reconstruction [Liu et al. 2011]) and chromoplexy (“pleko” for to braid or to weave [Baca et al. 2013]). Chromothripsis corresponds to the shattering of a chromosome, or a chromosomal region, into tens to hundreds of fragments, which are later reassembled in a random order and orientation, generally involving nonhomologous end joining (NHEJ) (Stephens et al. 2011; Koltsova et al. 2019; Brás et al. 2020; Dewhurst 2020; de Groot et al. 2023). Chromoanasynthesis occurs on a single chromosome due to error-prone DNA replication, leading to copy number variations (Liu et al. 2011; Ostapińska et al. 2022; Guo et al. 2023a). In contrast to the two aforementioned processes, chromoplexy involves multiple chromosomes with intra- and interchromosomal translocations (Baca et al. 2013; Guo et al. 2023a).

Several intrinsic and extrinsic factors have been implicated in the genesis of these catastrophic genomic events (Holland and Cleveland 2012; Koltsova et al. 2019; Pellestor and Gatinois 2020; Guo et al. 2023a). Endogenous mechanisms include cellular processes, such as micronucleation (Zhang et al. 2015), abortive apoptosis (Tubio and Estivill 2011), defects in the p53 pathway (Haferlach et al. 2008), and telomere crisis (Maciejowski et al. 2015). Additionally, environmental factors, such as exposure to ionizing radiation (Morishita et al. 2016), viral infections and genomic integration (Schütze et al. 2016; Koltsova et al. 2019), as well as artificial genome editing techniques such as Clustered Regularly Interspaced Short Palindromic Repeats and CRISPR-associated protein 9 (Leibowitz et al. 2021) or biolistic transformation (Liu et al. 2019), have also been demonstrated to contribute to these events.

Initially discovered in human cancer cells, chromoanagenesis has since been documented across diverse eukaryotes, including mammals (Villagómez et al. 2008; Pinton et al. 2009; Carbone et al. 2014; Guo et al. 2023a) and plants (Guo et al. 2021, 2023a, 2023b). While it is often deleterious in animals, such genome restructuring seems to be more tolerated in plants, where it may contribute to adaptation, gene innovation, and genome evolution (Pellestor and Gatinois 2020). Similar large-scale genomic alterations have also been reported in model organisms such as *Saccharomyces cerevisiae* (Anand et al. 2014; Marie-Nelly et al. 2014; Yue et al. 2017), *Caenorhabditis elegans* (Itani et al. 2016), as well as in the marine phytoplankton genus *Ostreococcus* (Blanc-Mathieu et al. 2017).

*Ostreococcus* is a genus of unicellular eukaryotic picoalgae (cell size <2 µm) from the order Mamiellales (Chlorophyta) with a cosmopolitan distribution in the sunlit ocean (Delmont et al. 2022; Yung et al. 2022). Its compact haploid 13 Mb genome makes it a valuable model for studying chromoanagenesis-like events. It contains 18 standard chromosomes and 2 so-called outlier chromosomes: the big outlier chromosome (BOC) and the small outlier chromosome (SOC) (Moreau et al. 2012). These chromosomes exhibit atypical features, low guanine and cytosine (GC) content, higher transposable element density, abundant repetitive sequences, and reduced gene density, relative to the rest of the genome (Moreau et al. 2012). While the BOC is the mating type determination candidate (Blanc-Mathieu et al. 2017), the SOC is thought to play a role in antiviral defense (Yau et al. 2016). The hypervariability of the SOC in natural populations, along with its mosaic structure, suggests that *Ostreococcus* undergoes—or tolerates—chromosomal reshuffling events reminiscent of chromoanagenesis (Blanc-Mathieu et al. 2017). This underscores its potential as a model for investigating the origin and consequences of chromosome instability in unicellular eukaryotes.

*Ostreococcus* is infected by prasinoviruses, which are double-stranded DNA viruses classified within the phylum *Nucleocytoviricota*, also known as nucleocytoplasmic large DNA viruses (NCLDVs), which are extremely abundant in their natural environment (Meng et al. 2021). These viruses replicate within the host, assembling in the cytoplasm and ultimately causing cell lysis (Dunigan et al. 2006; Derelle et al. 2008; Weynberg et al. 2017). Interestingly, *Ostreoccocus* populations develop reversible resistance to prasinoviruses, allowing for long-term coexistence (Yau et al. 2020). A population is considered susceptible (S) if a significant proportion of its cells can be infected, leading to lysis and viral production. However, after lysis, the population recovers systematically (Yau et al. 2016). Once a recovered population no longer undergoes lysis upon reexposure to the virus, it is considered resistant (R). Resistant populations primarily consist of uninfected R cells, although viral production still occurs at low levels, probably due to infection of a minority of susceptible cells forming a coexistence dynamic, akin to a bet-hedging strategy between susceptible and resistant cells (Yau et al. 2020). The molecular mechanisms underlying the emergence of antiviral resistance remain poorly understood. Previous work demonstrated the systematic evolution of resistance to viruses in *Ostreococcus tauri* and its association with karyotypic changes in the SOC (Yau et al. 2016, 2018). Intraspecific genome comparison revealed that the length of the SOC was positively correlated with resistance to a broader spectrum of prasinovirus strains (Blanc-Mathieu et al. 2017). Reciprocally, the transition from a resistant parental line to susceptible experimental evolution lines has been associated with large deletions on the SOC in the species *O. mediterraneus* (Yau et al. 2020). These previous results prompted us to reinvestigate the genomic bases of resistance acquisition in *O. mediterraneus.* To elucidate the genetic variation associated with the evolution of resistance to viruses in *O. mediterraneus,* we identified structural variations, as well as point mutations, linked to susceptible or resistant phenotypes, and the presence or absence of viral sequence insertion. Genomic analyses of five independent pairs of virus-susceptible control and evolved-resistant lines of *O. mediterraneus* revealed substantial structural variation within the SOC. Surprisingly, no evidence was found for an association between this variation and the resistant phenotype, while sequence analyses highlighted a high rate of spontaneous structural rearrangements relative to point mutations in this chromosome.

## Results

### Systematic Evolution of Resistance to Prasinovirus OmV2 in Ostreococcus Mediterraneus

**Fig. 1.**
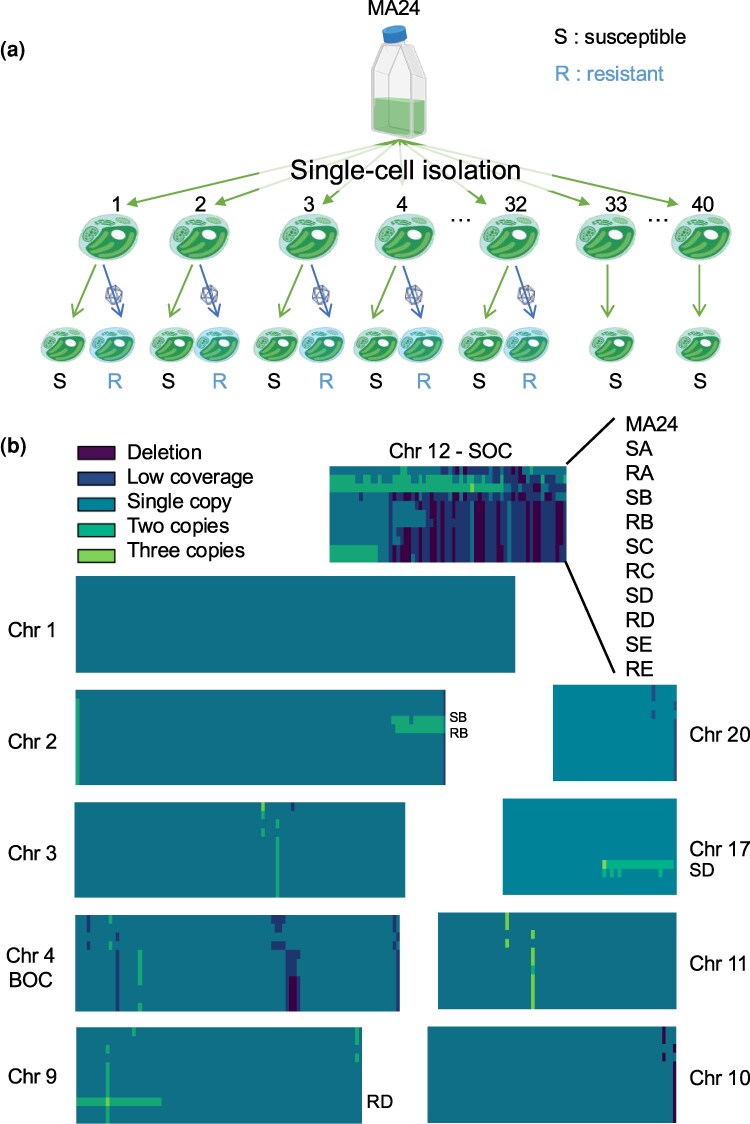
Detection of copy number variations in the genome of *O. mediterraneus* from virus-susceptible and resistant experimental evolution lines. a) Illustration of the experimental evolution study setup. Single-cells from the parent virus-susceptible line (MA24) were sorted into 40 culture lines, 32 of which were divided into pairs to which the prasinovirus OmV2 was added to one of each pair. All lines challenged with OmV2 lysed, regrew, and developed resistance. b) Genome-wide copy number variations in the parent MA24 and the five experimental evolution lines analyzed, comprising virus-susceptible (S) and virus-resistant (R) pairs with each line designated as A to E. The data from each experimental line were stacked for each chromosome. Copy number variations were estimated by short-read mapping onto the reference *O. mediterraneus* RCC2590 genome, with the read coverage of each chromosome normalized to the coverage of chromosome 5. Coverage was estimated over 10 kb windows. The 10 chromosomes with the most copy number variations and chromosome 1 with no variations are displayed (the complete heatmaps are available in Fig. S1). Specific lines with large copy number variations in chromosomes 2, 9, and 17 are labeled.

To understand the genomic basis of resistance acquisition to prasinovirus, an experimental evolution study was conducted with 40 independent culture lines, derived from a single cell of the virus-susceptible *O. mediterraneus* strain MA24 (Yau et al. 2020). Of these, 32 lines were split into infected and uninfected control flasks, while 8 lines remained as uninfected controls (Fig. 1a). All infected cultures visibly lysed within 3 d post-infection, but resistance to prasinovirus systematically evolved in all 32 infected lines, as shown by regrowth within 5 d postinfection. Whole-genome sequences of five selected pairs of resistant (R) and susceptible control (S) lines, designated lines A to E, were obtained using Illumina short-read and Oxford Nanopore Technology long-read sequencing.

### Line-Specific and Shared Copy Number Variations on Standard Chromosomes

The experimental evolution lines (A to E) and the parental line (MA24) were screened for copy number variations relative to the ancestral reference line RCC2590 by analyzing chromosome coverage in 10 kb windows using the Illumina short reads. Most of the 20 chromosomes showed no large copy number variations (Figs. S1 and S2). The only copy number variation consistently shared across all five R and S line pairs was a 10-kb duplication at the left end of chromosome 2 (Fig. 1b). Beyond this shared event, seven standard chromosomes exhibited notable copy number variations, most prominently chromosomes 2, 9, and 17 (Fig. 1b). On chromosome 2, both B line strains (SB and RB) carried a 120-kb duplication at the right end of the chromosome. Notably, the D lines displayed two large copy number variations >100 kb: strain RD had a 240-kb duplication at the end of chromosome 9, including a 10-kb triplication, while strain SD carried a 170 kb duplication at the end of chromosome 17. Interestingly, this same region of chromosome 17 shows slightly increased coverage in strain RD (Fig. S2), suggesting that a subset of cells also harbor this duplication. Apart from the aforementioned copy number variations, the R and S strains in lines C, D, and E exhibited similar copy number variation patterns across the genome. Likewise, SA and SB showed shared copy number variations and overlapped significantly with those found in the parental line MA24. However, the most substantial copy number variations, both deletions and duplications, across all strains were concentrated on a single chromosome: chromosome 12, which is SOC in this species.

### The SOC as a Hotspot of Line-Specific Copy Number Variations

**Fig. 2.**
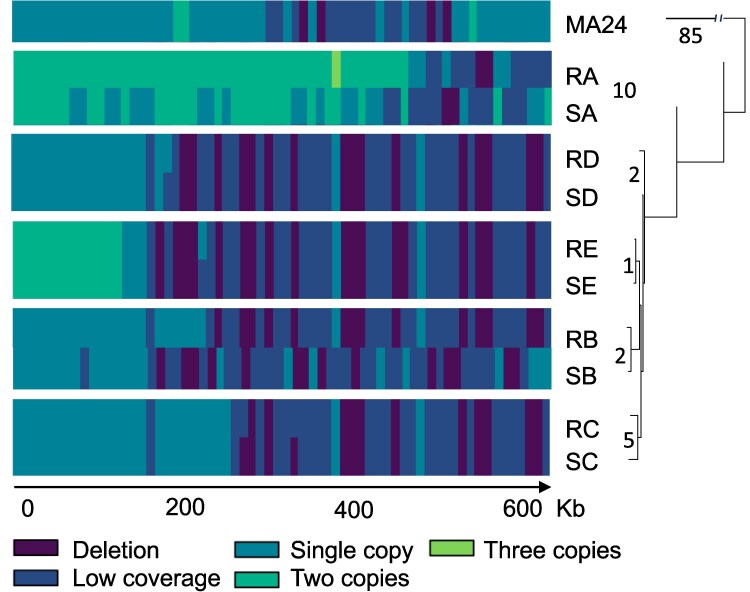
Copy number variation along the SOC compared with the fixed mutations on the standard chromosome in each line. Left: A zoomed-in view of the SOC coverage from Fig. 1 showing deletions and duplications relative to the 653-kb reference genome. Right: Dendrogram showing the clustering of the 11 strains based on 111 SNPs detected in the callable genome, excluding the SOC (Table S3). The numbers indicate the ancestral-specific mutations (present only in MA24) and the mutations distinguishing each pair of strains (A to E).

Most copy number variations between strains were concentrated on the SOC. A striking observation was the presence of large low coverage blocks spanning ∼400 kb region at one end of the SOC, indicating extensive deletions concentrated in this region across all lines. We compared the size and positions of these deletions in the parental line MA24 and the ten experimental evolution lines relative to the 637-kb SOC of the reference strain RCC2590. MA24 exhibited a 220-kb deletion between positions 300 to 520 kb. Interestingly, portions of this deleted region in MA24 were retained in all experimental evolution lines, particularly in line A. This pattern suggests a deletion polymorphism in the parental population, which was later differentially assorted among experimental evolution lines. Supporting this, pulsed-field gel electrophoresis from Yau et al. (2020) showed that MA24 harbored at least two SOC variants, one ∼453 kb and another around 200 kb in size, consistent with the presence of two SOC deletion variants in the parental MA24 population. Thus, strain RA, which had the smallest deletion (∼150 kb, Fig. 2), likely derived from a lower frequent SOC variant in the MA24 population. In contrast, the largest deletion (∼480 kb) was found in the E strains, suggesting an additional SOC deletion event occurred after the divergence from the parental line.

The second striking observation was a duplication at the left end of the SOC in strains A and E. In the A strains, nearly the entire 470 kb region at that end was duplicated, with a segment between positions 380 to 390 kb in RA suggesting a triplication. In the E strains, a 130-kb region at the same end was duplicated. Additionally, SOC coverage patterns revealed that independent copy number variations occurred between the resistant and susceptible members of each A to E line pair, suggesting these rearrangements likely arose after the evolution of resistance to prasinovirus OmV2. Despite this, SOC coverage profiles indicated that strains primarily clustered by the experimental lineage map (Fig. 1a), and no SOC variant could be consistently associated with either the resistant or susceptible phenotype.

### Single Nucleotide Variation Reflects the Experimental Lineage Map, Not Resistance to OmV2

To investigate whether any SNPs were associated with the R or S phenotypes, we identified SNPs by mapping Illumina short reads from each line to the RCC2590 reference genome. A total of 120 SNPs were located within the callable genome, defined as regions covered in all 10 strains and thus excluding the highly repetitive SOC (see Materials and Methods). The number of SNPs per line ranged from 66 in line RC to 86 in line SC (Table S1), with 18 SNPs detected in the parental line MA24. Within pairs, the number of SNP differences varied from just one SNP in the SE/RE pair to as many as 10 between SA and RA. A phylogenetic tree based on SNP distances largely reflected the experimental lineage map, with S and R pairs clustering together rather than by phenotype, except for strain RA, which diverged from this pattern (Fig. 2). Importantly, no SNPs were found to be uniquely associated with either the R or the S phenotype on the callable genome. To test whether there was an elevated mutation rate in any of the lines, we estimated the number of segregating SNPs in each dataset (Table S1). There was no significant difference in the number of segregating SNPs between the R and S phenotypes (Student’s *t-*test *P* = 0.70).

In conclusion, neither SOC copy number variants nor SNP distributions could be directly associated with the development of resistance to prasinovirus OmV2. Instead, we observed large insertions and deletions suggestive of spontaneous structural variations of the SOC, occurring independently of viral infection and acquisition of resistance.

### Hybrid Genome Assemblies Resolve Structural Variants Predicted by Copy Number Variation

**Fig. 3.**
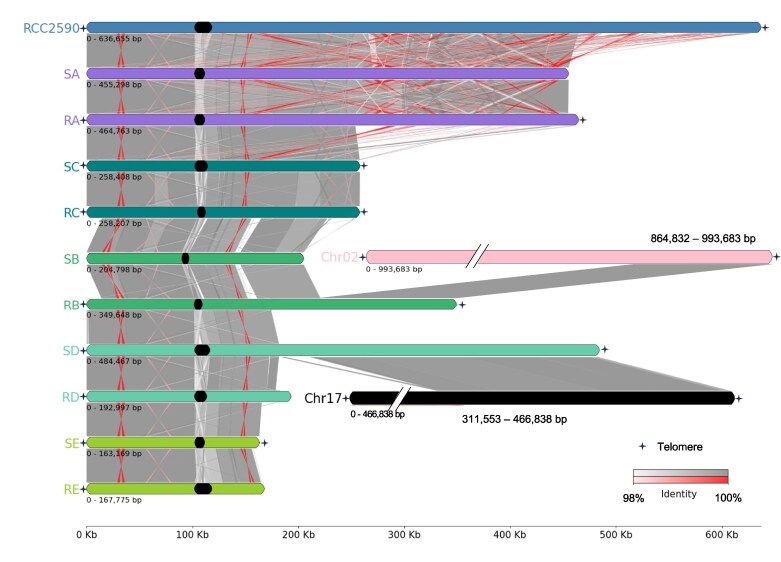
Comparison of the SOC assemblies of the experimental evolution lines and the reference RCC2590. High nucleotide identity (98% to 100%) regions between pairs of chromosome assemblies are joined with shaded gray (same orientation) and red (reverse orientation) blocks. SOC assemblies from the same experimental evolution line pair (R/S) share the same color. Interchromosomal translocations are shown by alignment of the SOC to the reference chromosome 2 (Chr02) and chromosome 17 (Chr17). The black rectangles on the SOC assemblies show the size and location of a repeat region.

To more precisely characterize these structural variations, all SOCs were reconstructed using hybrid de novo assemblies combining long- and short-read sequencing, yielding complete or near-complete chromosomal assemblies (Fig. 3). All generated Masurca-Flye assemblies showed high gene completeness (BUSCO 95.8% to 97.1%) and excellent base accuracy (QV score 49.8 to 57.7) (Table S2). Each SOC assembly was validated by remapping both short and long reads to confirm sequence coverage and scaffold placement (Table 1). The SOCs of the 10 experimental evolution lines grouped into four distinct structural types.

**Table 1:** Assembly features of each SOC

Strain line	Name of contigs	RCC2590 order	Telomeres	Bridging long reads
SA	Reverse AP0970.masurca_flye2.5_s24	1	0	158
	AP0970.masurca_flye2.5_s22	2	0	
SB	Reverse AP0971.masurca_flye2.5_s098	1	0	20
	AP0971.masurca_flye2.5_s063	2	0	19
	Reverse AP0971.masurca_flye2.5_s106	3	0	10
	Reverse AP0971.masurca_flye2.5_s093	4	0	4
	AP0971.masurca_flye2.5_s072	5	0	
SC	AP0974.masurca_flye2.5_s25	1	1	42
	AP0974.masurca_flye2.5_s24	2	1	
SD	AP0967.masurca_flye2.5_s15	1	2	35
SE	AP0976.masurca_flye2.5_s31	1	1	124
	AP0976.masurca_flye2.5_s34	2	1	
RA	AP0969_SOC	1	2	-
RB	AP0972.masurca_flye2.5_s13	1	2	19
RC	Reverse AP0973.masurca_flye2.5_s50	1	1	26
	AP0973.masurca_flye2.5_s60	2	0	
	AP0973.masurca_flye2.5_s52	3	0	28
	Reverse AP0973.masurca_flye2.5_s76	4	0	27
	Reverse AP0973.masurca_flye2.5_s65	5	1	20
RD	Reverse AP0968.masurca_flye2.5_s29	1	1	
	Reverse AP0968.masurca_flye2.5_s33	2	0	29
	Reverse AP0968.masurca_flye2.5_s42	3	0	16
RE	AP0975.masurca_flye2.5_s25	1	1	18
	Reverse AP0975.masurca_flye2.5_s33	2	0	

The first type of assembly spanned the entire chromosome from telomere-to-telomere and included the SC and RC lines, as well as RA and SE. These SOCs exhibited a large deletion at one end compared with the reference, consistent with previous coverage analyses. Their sizes were 258, 258.5, 426, and 163 kb, respectively, much shorter than the length of the 637-kb reference SOC.

The second type comprised SOCs with a single telomere (RD and RE). The SOCs of RD and RE (193 and 168 kb long, respectively) contained a telomere at the left end and exhibited large deletions of 444 and 470 kb at the right end, also consistent with the coverage analyses.

The third assembly type corresponded to the reconstructed SOC of line SA and SB, which lacked identifiable telomeric repeats. SB has a size of 205 kb, presenting a deletion of 15 kb on the left end and one of 417 kb at the right end. For SA, despite the absence of telomeres, the left end was identical to those of other SOC assemblies, and the right end exhibited a similar large deletion like its paired line, RA. This was consistent with coverage analyses, suggesting that the SA SOC assembly was nearly complete. The final estimated size was 455 kb, with a 145-kb deletion at the right end.

The fourth type of assembly included the SOCs of strains RB and SD, both of which formed telomere-to-telomere assemblies that, unexpectedly, incorporated sequences from another chromosome. In both cases, the left end of the chromosome was from the SOC. In strain RB, the assembly joined 220 kb of the SOC with a duplicated and translocated 120 kb segment from the right end of chromosome 2. Coverage analyses confirmed this duplication on chromosome 2 (Fig. 1b). In strain SD, 181 kb of the SOC's left end was joined to chromosome 17. Both chromosome fusions are validated by short-read and long-read alignments on the fusion site (Fig. S3) and by both the Masurca-Flye and the Canu-Racon assemblers (Fig. S4). In SD, the Canu-Racon assembly fusion suggested a SOC-ch17 fusion with the duplicated region of ch17, whereas the Masurca-Flye assembly suggested a fusion of the SOC with the single copy of ch17. These two configurations are equally plausible because the fusion includes a repeated region in ch17, which exists in both direct repeat version (as in the reference ch17 and the Canu-Racon ch17 assembly) and inverse repeat configuration (in the Canu-Racon SOC assembly). No long-read sequences merge the SOC to the second duplicated region, so that we lack data available to validate one configuration over the other. However, since the Canu-Racon assembly corresponds to the most parsimonious karyotypic change, one that leaves the standard ch17 intact, we consider the SOC assembly suggested by Canu-Racon the most likely. We also inspected the two predicted fusion sites for microhomologies or repeated elements. A 4-nucleotide microhomology was identified at the SOC-ch02 fusion site in RB, while the SOC-ch17 fusion site in SD lacked any specific pattern aside from chromosome-specific repeated sequences within ±10 kb of the junction (Fig. S5). These observations support the potential involvement of NHEJ in these translocations.

There is, however, one discrepancy between the coverage analyses and the de novo assemblies concerning the 5′ duplications observed in lines A and E: the size of the duplicated part identified in the coverage analyses closely matches the size of the respective SOC assemblies. As no other chromosomes in these lines showed evidence of duplication, we inferred that lines A and E each harbor two copies of the SOC.

### Conservation of OmV2 Insertions Across Experimental Evolution Lines

Given recent evidence of endogenous viral elements in algal genomes (Moniruzzaman et al. 2020; Gyaltshen et al. 2023; Erazo-Garcia et al. 2025), the genome assemblies of the reference and the 10 evolution experimental lines were screened for insertion of DNA from the OmV2 virus. In the reference genome (RCC2590), five OmV2-derived insertions were identified, located on chromosome 4 (73 bp), chromosome 6 (58 bp), chromosome 8 (80 bp), chromosome 10 (36 bp), and chromosome 11 (339 bp). These five insertions were conserved across all experimental lines, except for the chromosome 4 OmV2-derived insertion in SB. No new OmV2 insertions were identified in any of the 10 experimental evolution lines.

### Accordion-Like Dynamics of Polymorphic Tandem Repeats

**Fig. 4.**
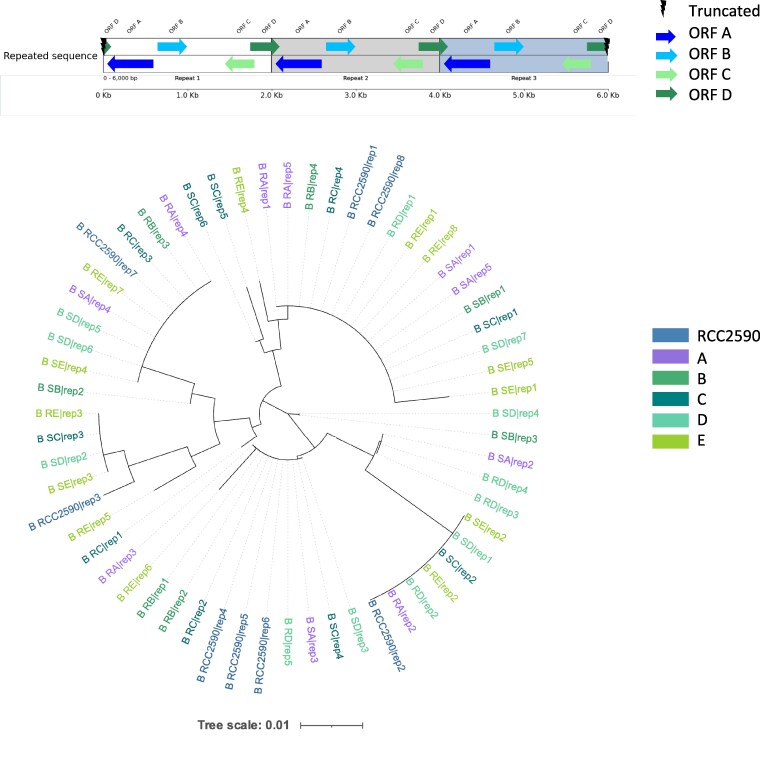
Features of the tandem repeat “accordion” region and inference of its evolutionary history in the experimental evolution and reference lines. a) Schematic representation of three copies of the accordion region showing the relative positions of predicted ORFs. b) Phylogenetic tree using neighbor-joining of the 114 amino acids of all the copies of ORF B in the 11 lines (experimental evolution lines A to E and reference RCC2590). The red branch line highlights the only identical copy present in all strains.

One region of the SOC assemblies, consisting of three to eight tandem repeats of an ∼1.8 kb unit (Fig. 4) presented exceptional dynamics. The minimal structural rearrangement of this region observed within a pair of lines was observed in RA and SA with an identical number of five repeats. A similar region had previously been identified in the SOC of a natural *O. tauri* population (Yau et al. 2016; Blanc-Mathieu et al. 2017). However, there was no significant local alignment between the *O. tauri* and *O. mediterraneu*s repeats. The repeats occurred in tandem (Fig. 4), with polymorphisms among copies. Repeat sizes ranged from 1,685 to 1,985 bp, with size variation primarily due to small terminal deletions on the final repeat, with the terminal repeat in line RD truncated to 777 bp.

Some of the repeats were interrupted by a 1,001-bp spacer sequence, increasing the total size of the composite repeat unit to 2,929 bp. This spacer contained long inverted repeats of 225 bp but did not encode any identifiable open reading frames (ORFs). The same spacer sequence was detected six times in the reference genome—on chromosomes 4, 7, 8, 9, 10, and within the SOC. Within the polymorphic tandem repeat regions, the spacer was present only once per array. Although the spacer appeared in the consensus sequences of lines SA, SB, SC, RD, and RE, long-read analysis revealed its presence in all SOC assemblies except that of line SD. The position of the spacer-containing repeat varied, with one to three possible insertion sites across the population, accounting for its absence in some consensus sequences. Based on its consistent presence and polymorphic position, this spacer may represent a nonautonomous transposable element that inserted prior to the expansion of the tandem repeat arrays. Its inactivity is suggested by the constant single copy of the spacer, despite variation in repeat number through duplication or deletion.

During this study, the various sequence similarity searches (see Materials and Methods) of the entire polymorphic tandem repeated regions, or the repeats themselves, did not allow the identification of any similar sequences in GenBank (nr, nr/nt), suggesting these repeats are fast evolving and specific to *O. mediterraneus*.

Candidate ORFs were detected within all the polymorphic tandem repeated regions. None of these ORFs contained known functional domains, as determined by InterProScan analysis. The ORFs were designated A, B, C, and D according to their relative positions within the repeat unit (Fig. 4a). An additional reverse ORF, named E, was identified in the SD line, located between the ORFs B and C. Each of the four primary ORF groups included both a more conserved form and variants characterized by point mutations and indels.

Further protein structure predictions suggested that ORFs C and E may contain a transmembrane domain. For the remaining ORFs, structural predictions were inconclusive. Notably, a gene annotated in the *O. mediterraneus* genome (Ostme30g0062), composed of two exons and one intron, corresponds in both sequence and genomic position to a combination of ORF C and E. The predicted structure of Ostme30g0062 matches the structure of a predicted monomeric transmembrane protein of *Micromonas commoda* and of a predicted monomer forming the trimeric transmembrane protein in *Ostreococcus lucimarinus* and *Micromonas pusilla*. This structure conservation suggests that the sequence may play a functional role shared among related marine algae.

Since ORF B was the only ORF consistently present in all but the truncated terminal repeat, it was used to investigate the evolutionary history of the tandem repeats within and between the strains. An alignment of the 114 amino acids of the ORF B copies from all strains was used to construct a phylogenetic tree (Fig. 4b). The resulting phylogeny revealed that copies from all the strains had mixed origins, with some likely inherited vertically and others having diversified independently within specific lineages. Only one ORF B copy was identical across all strains (highlighted in red in Fig. 4b); however, this copy did not occupy the same position in the repeat array in each strain, indicating polymorphic rearrangements in repeat order among strains. Phylogenetic trees constructed from alignments of the most conserved versions of ORFs A, C, and D supported this pattern of strain-specific expansion, contraction, and rearrangement of the repeat structures.

To assess whether the predicted ORFs in the repeat units were likely to encode functional proteins under selection on amino-acid composition, we estimated the number of synonymous and nonsynonymous mutations among ORF copies (Table S3). These analyses did not allow us to reject the null hypothesis of an equal rate of synonymous and nonsynonymous changes, consistent with a lack of selection on the amino-acid sequences of these putative ORFs. Therefore, ORFs A, B, C, and D are unlikely to encode protein-coding sequences.

Overall, this region was shown to be highly dynamic, with lineage-specific repeats with modifications suggesting a high point mutation rate, as well as cycles of expansion and contraction—an accordion-like mechanism shaping repeat copy number and order.

## Discussion

### Structural Variations Point Toward Unique Chromosome Instability in One Chromosome

We resolved large structural variations and point mutations in the genomes of both susceptible and resistant experimental evolution lines. The dynamics of chromosome 12, the SOC, stood out, as it exhibited the most extreme variation in read coverage among strains. Large-scale deletions at its 3′ end were observed across all lines, confirming previous observations based on pulsed-field gel electrophoresis and hybridization (Yau et al. 2020). The ten outlier chromosome assemblies revealed large deletions and duplications, including whole-chromosome duplications, intra- and interchromosomal translocations, resulting in unique SOC assemblies for each of the ten lines.

The SOC has been previously characterized in all complete genome sequences of Mamiellales sequenced to date (Derelle et al. 2006; Worden et al. 2009; Moreau et al. 2012), whose species divergence is estimated to date back more than 300 million years (Yung et al. 2022), and aneuploidies involving the SOC have been previously reported in mutation accumulation lines of *Micromonas* and *Bathycoccus* (Krasovec et al. 2023). Additionally, population genomic analyses in one species revealed a striking contrast: whereas standard chromosomes show ∼1% nucleotide divergence between any pair of individuals, only 15% to 30% of the SOC chromosome could be aligned between individuals from the same population (Blanc-Mathieu et al. 2017).

While this novel type of hypervariability was proposed to result from a chromothripsis-like process (Blanc-Mathieu et al. 2017), the structural rearrangements we describe here are not consistent with chromothripsis as the evolutionary mechanism for this chromosome. Instead, the process resembles partial chromosome duplication and chromoplexy, involving only a few catastrophic events rather than the chromosome shattering characteristic of chromothripsis (Guo, Comai and Henry 2023a).

### Large Chromosome Duplications and Chromoplexy Drives Diversification of the SOC

The high rate of duplication, deletion, and interchromosomal fusion observed on this chromosome within the ∼190 generations separating each pair of lines thus helps us better understand the evolutionary dynamics of this outlier chromosome.

**Fig. 5.**
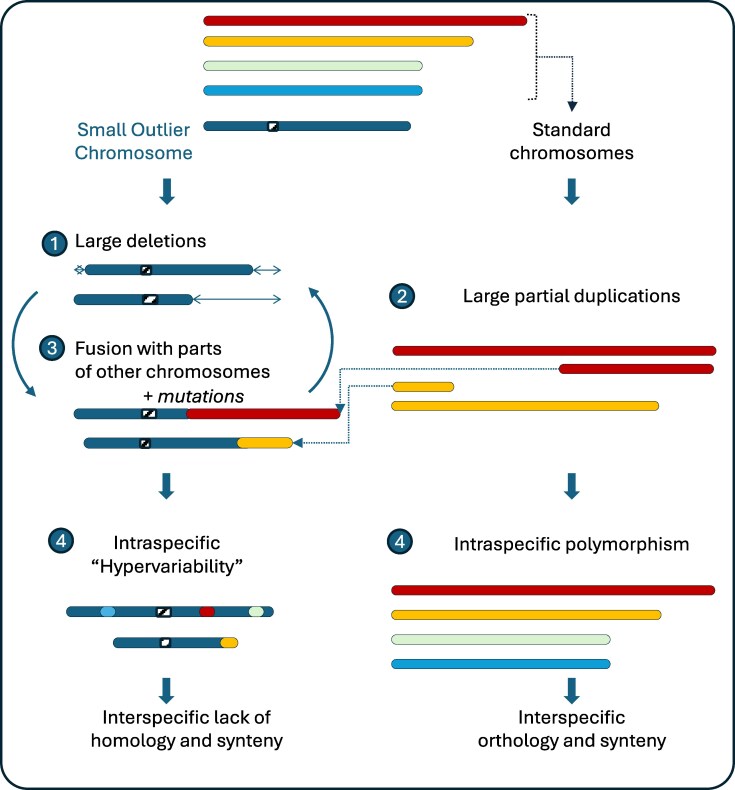
Model of hypervariable chromosome evolution driven by large deletions, fusions with parts of duplicated chromosomes, and mutations (left part), as opposed to the evolution of standard chromosomes (right part).

First, chromoplexy explains the high number of strain-specific genes on this chromosome, ie genes uniquely present in one strain and not in other strains from the same species (Blanc-Mathieu et al. 2017). Indeed, frequent random fusion with parts of different chromosomes, followed by deletions, causes rapid diversification of the gene content between strains on this chromosome within a population. Using this new information about the origin of the SOC, we specifically searched for sequence homologies between the standard chromosomes and the SOC, while excluding repeated elements that occur in multiple genomic locations. This approach led to the identification of 25 signatures of past translocation events, comprising a total of 77 Kb within the SOC that share significant sequence identity with regions of the standard chromosomes. Notably, this includes a 36-Kb segment originating from chromosome 4. On the longer timescale of speciation, chromoplexy is thus expected to gradually lead to the loss of synteny on this chromosome (Fig. 5), which has been reported in early comparative genome studies in *Ostreococcus* and *Bathycoccus* (Palenik et al. 2007; Moreau et al. 2012).

More precisely, the present experiment provides insights into the relative tempo of structural variation on this chromosome versus point mutations on the standard chromosomes: during the course of these five independent infection and resistance evolution experiments, the ten strains have accumulated between one and ten independent point mutations on standard chromosomes between pairs (Fig. 2). These can be compared with the total number of independent structural mutations on the SOC. Structural variations between each pair of strains corresponded to a minimum of—counting the minimum number of indels and accordion repeat copy number variations—ten duplications-deletions larger than 10 kb and two large chromosomal translocations from chromosome 17 (300 kb) and 2 (120 kb). Therefore, the number of large structural variations (12) on this chromosome is of the same order of magnitude as the number of point mutations on all 18 standard chromosomes (20). The spontaneous mutation rate in the smallest photosynthetic eukaryote corresponds to approximately one point mutation every 100 cell divisions (Krasovec et al. 2017). This, in turn, allows us to calibrate the structural divergence observed between two strains with the number of nucleotide differences we observe between two strains. Under the hypothesis that the structural variations of the SOC observed in *O. mediterraneus* can be extended to other Mamiellales species, the 50,000 point mutations between two pairs of strains in a natural population of *O. tauri* (Blanc-Mathieu et al. 2017) would correspond to approximately the same number of structural changes on the SOC. This is consistent with the low level of synteny on the SOC among closely related strains. Because of the high divergence time between each Mamiellales species sequenced so far and the saturation of substitutions at synonymous positions, equivalent to more than one substitution at each neutral site, this relative timeline also accounts for the complete lack of synteny on this chromosome observed between species (Palenik et al. 2007; Worden et al. 2009; Moreau et al. 2012).

### Sequence Divergence in Genes Located on the SOC

Another feature of the SOC is the sharp decrease in the proportion of orthologous genes located on this chromosome (Jancek et al. 2008). This pattern could be explained by three, nonmutually exclusive, scenarios: (i) a high turnover of gene content driven by the integration of sequences from other lineages, such as viruses; (ii) an elevated point mutation rate obscuring sequence identity; or (iii) an elevated fixation rate of mutations as a consequence of reduced recombination. These three possibilities are discussed in detail below.

Indeed, green algae frequently show evidence of viral sequence insertions, including from giant viruses (NCLDVs) and virophages (Sheng et al. 2022), with some integrations being quite extensive (Moniruzzaman et al. 2020). However, no newly inserted OmV2 sequences were detected during this experiment. This argues against the hypothesis of the SOC being a hotspot for ongoing viral integration—at least in *O. mediterraneus*. It suggests instead that prasinoviruses either rarely integrate into this genome, or that viral sequences are efficiently eliminated following genome integration.

Nevertheless, viral-derived sequences are not entirely absent. Previous studies identified viral segments in both the standard and SOC of *O. tauri* (Blanc-Mathieu et al. 2017), and we also detected short prasinovirus-like sequences within the standard chromosomes of *O. mediterraneus*. While these fragments are limited, their presence suggests ancient or low-frequency integration events, even if the SOC is not an active hotspot of viral integration in this species.

Thus, an elevated point mutation rate on genes on the SOC seems the more likely explanation for the low rate of orthologous relationships on genes located on this chromosome. Estimating the point mutation rate is notoriously difficult in highly repetitive regions, which are typically excluded from spontaneous mutations estimates (Krasovec et al. 2017, 2023). However, our analysis of the polymorphism of the accordion region suggests many more point mutations occur within this region than within the standard chromosomes, and this would be consistent with an elevated nucleotide mutation rate on specific regions of this chromosome. Another plausible evolutionary scenario over a longer timescale is that the lack of recombination in such rearranged regions promotes the evolution of genomic islands of divergence, a pattern that has been reported in many species (Sodeland et al. 2016; Dobry et al. 2023; Marín-García et al. 2024; Akopyan et al. 2025).

### Are Chromosome Copy Number Variation, Chromoplexy, and Transcriptional Reprogramming of the SOC Connected?

In the nucleus of eukaryotic cells, chromosomes are folded into subnuclear domains known as chromosome territories (Cremer et al. 2006; Meldi and Brickner 2011). This folding is dynamic, allowing for the formation of chromatin loops that facilitate both inter- and intra-chromosomal interactions involved in gene coregulation (Simonis et al. 2006; Meldi and Brickner 2011). The spatial positioning of chromatin within the nucleus influences transcriptional activity: repressed regions are often localized at the nuclear periphery, while reactivation is associated with their relocation to the nucleoplasm (Ragoczy et al. 2003; Reddy et al. 2008; Meldi and Brickner 2011). The SOC has a bipartite transcriptional organization (Yau et al. 2016), with the accordion region demarcating the two parts of the chromosomes being transcriptionally active or inactive in different strains. The SOC may therefore have up to 80% of its chromatin in a transcriptionally repressed state in some strains (Yau et al. 2016). Therefore, a large part of the nontranscribed SOC is expected to be located within the nuclear periphery and membrane.

This, in turn, may contribute to a higher rate of structural variation, as impaired migration during mitosis can lead to micronucleus formation. Chromosomes trapped within micronuclei are exposed to a more damaging environment, making them more susceptible to chromoanagenesis (Leibowitz et al. 2015; Koltsova et al. 2019; Guo et al. 2023a). Additionally, the formation of chromatin loops extending beyond chromosome territories for interchromosomal gene coregulation may facilitate interchromosomal rearrangements. To date, micronuclei have never been reported in these species. However, recent chromosome Hi-C analyses indeed suggest an insulated position for the SOC in *O. tauri (Valiadi et al. 2025).* Therefore, a deeper understanding of nuclear organization is poised to provide valuable insights into genome plasticity, particularly concerning the unique characteristics of the SOC.

### A Highly Polymorphic Accordion Region Lacking Conclusive Evidence of Protein-Coding Potential

While the structural rearrangements underlying both intraspecific hypervariability and interspecific synteny loss have now been characterized, the role of this chromosome in conferring resistance to prasinoviruses remains unclear. Our results demonstrate that chromosomal rearrangements occur independently of viral infection. Although we extended previous observations of systematic resistance evolution following viral infection in O. *tauri* (Yau et al. 2016) to *O. mediterraneus*, we did not identify any common genome rearrangement associated with the five resistant strains. The only systematic changes between S and R were in the sequence of the SOC and the accordion region, either in the number of tandem repeats or in mutations affecting the number of predicted ORFs.

The core of the accordion region consists of a long polymorphic sequence repeated in tandem, with copy numbers ranging from 3 to 8. However, the number of copies does not appear to correlate with the resistance phenotype: one lineage (A) had identical copy numbers in S and R strains, two lineages (B and E) had more copies in R strains, while two others (C and D) had more in S strains. If gene copy number in this region is associated with the phenotype, it is unclear whether these genes encode for protein-coding genes or noncoding RNAs. Among the ORFs identified in the accordion region, ORF B was consistently found across all strains and all repeat copies, yet it shares no homology to any known protein-coding gene.

Interestingly, the only open reading frame in the accordion region with a predicted protein structure is a fusion of ORFs C and E, which likely encodes a transmembrane protein (Ostme30g0062). As homologous genes can be identified in three other species, this open reading frame likely encodes a protein sequence.

The lack of evidence for other protein-coding genes for the ORF in this region points toward the encoding of functional alternatives such as noncoding RNAs. Noncoding RNA longer than 200 nucleotides (lncRNAs) that do not code for proteins, typically evolve rapidly, show low sequence conservation, and are often expressed in a highly context-specific manner. Their evolutionary dynamics are strongly influenced by genomic features such as bidirectional promoters and transposable elements, both of which are known to facilitate the gain and loss of lncRNA loci (Kapusta and Feschotte 2014). As such, genomes rich in transposable elements tend to produce more flexible and rapidly evolving transcriptomes. In the nucleus, lncRNAs can modulate chromatin structure by interacting with chromatin-remodeling complexes, either by guiding, scaffolding, or sequestering them to specific genomic loci (Han and Chang 2015; Neve et al. 2021). LncRNAs are thus good candidates to be encoded within the transcriptionally dynamic SOC, and especially in the transcriptomic rewiring around the accordion region, which corresponds to the physical limit between repressed and expressed regions of the SOC (Yau et al. 2016).

In conclusion, we resolved the structural dynamics of the SOC, characterized by spontaneous extensive tandem repeat polymorphisms and structural rearrangements, including translocations from other chromosomes. The inherent instability of the SOC highlights its potential as a key player in fast evolution, raising intriguing questions about the molecular mechanisms involved and the broader implications for the origin and resolution of spontaneous complex chromosomal rearrangements in eukaryotes. Future studies focusing on transcriptomic and proteomic analyses, as well as chromatin spatial organization, are poised to shed more light on the molecular mechanisms underlying the relationship between transcription, structural variation, and viral resistance in *Ostreococcus mediterraneus*.

## Materials and Methods

### Algal Strains, Culture Conditions, and Experimental Evolution Experiment

In this study, all the strains belong to the species *O. mediterraneus*. The parent line of all strains, “MA24”, (S3 line in Yau et al. 2020) was obtained by mutation accumulation on the reference strain RCC2590 (Krasovec et al. 2017). Strains were maintained in culture in L1 medium prepared using autoclaved seawater, filtered at 0.22 µm. They were kept under 12:12 h light/dark regime using white light at 15 °C. Forty cultures of MA24 were grown from a single cell of the MA24 strain following the protocol described in Krasovec et al. (2016). Thirty-two cultures were divided into two flasks and one flask of each strain was infected with the prasinovirus OmV2, following the infection procedure previously described in Yau et al. 2020. After cell lysis, resistance to OmV2 developed in all infected cultures within 21 d. Eighteen resistant cultures survived until sequencing. Thus, 18 pairs of susceptible and resistant-evolved cultures were available, with independent evolution over 84 d since the viral infection (9 September 2019 to 2 December 2019). They were referred to as S and R in the manuscript. The study focuses on five pairs of R and S culture lines which developed resistance to the viruses, namely lines 6, 10, 14, 26, and 28, renamed A, B, C, D, and E, respectively, which have been sequenced on Illumina and Nanopore platforms. These lines were chosen based on a preliminary Pulsed Field Gel Eletrophoresis analysis confirming species identity and suggesting some karyotypic variation, consistent with those previously reported in *O. tauri* (Yau et al. 2016) *and O. mediterraneus* (Yau et al. 2020).

### DNA Extraction and Sequencing

DNA extraction and sequencing were performed as described in Thomy et al. (2021) . Briefly, DNA was extracted from five strains (A to E) for both phenotypes using the cetyltrimethylammonium bromide protocol. DNA quality was assessed using the absorbance ratios at 260/280 nm and 260/230 nm (NanoDrop), visualized by gel electrophoresis on a 0.8% agarose gel, and quantified by fluorometry (Quantus). The library was prepared using a modified KAPA HyperPrep kit protocol and then quality checked and quantified. Illumina and Oxford Nanopore Technologies sequencing were conducted on the 10 samples as described in Thomy et al. (2021). The Illumina dataset of MA24 was downloaded from GenBank (SRX10812426).

### Genome Assemblies and SOC Reconstruction

Raw Illumina paired-end libraries and Oxford Nanopore Technologies reads were used with MaSuRCa v3.4.1 (Zimin et al. 2013) and Flye v2.5 (Kolmogorov et al. 2019), which respectively construct mega-reads and assemble them, to obtain hybrid assembly.

To improve the assembly of the SOC, contigs belonging to the SOC (completely or partially) were identified using nucmer (mummer-4.0.0beta2), mummerplot and show-coords (Marçais et al. 2018). The identified contigs were used to reconstruct the chromosome with the RCC2590 SOC as reference (Geneious Prime 2023.0.4, https://www.geneious.com). Without overlap, the contigs were placed using the reference for orientation, thus ensuring a conservative estimation of the structural variation within the data. The reconstructions were validated through visual inspection using the Integrative Genomics Viewer (Robinson et al. 2011) of both long-read and short-read alignments (Fig. S5), resulting in high-confidence assemblies. The features of the SOC reconstruction are summarized in Table 1. To cross-validate the predicted chromosome fusions of the SOC in RB and SD, we performed an alternative assembly using Canu (Koren et al. 2017) and Racon (Vaser et al. 2017) (minReadLength=1000, minOverlapLength=500, correctedErrorRate=0.105, useGrid=false).

The presence of virus sequences in the SOC's assemblies was checked by BLASTing the OmV2 genome against the assemblies. To visually check the structural rearrangements and avoid false positive structural variants (David et al. 2024), the reconstructed SOCs were aligned against each other with blastn (length_thr=600, identity_thr=98) and the identity between strains was represented using PyGenomeViz (Shimoyama 2024) within a range of 98% to 100%. Lower identity was considered as nonconserved sequences.

### Structural Variations: Read Coverage Analyses

The short reads were mapped against the RCC2590 reference genome using bwa-mem2 (version 2.2, mem) (Vasimuddin et al. 2019). The coverage for windows of 10 kb was obtained with SAMtools (samtools-1.9, view, sort, and index) and BEDtools (bedtools2-2.18.0, makewindows and coverage). Read coverage per genome varied from 25× (MA24) to 705× (SA) (Table S5). To estimate copy number variation within and between genomes, we normalized the coverage of each region by the average coverage of chromosome 5 for each line, as it was consistently present as a single-copy chromosome across all 11 lines (Figs. S2 and S6). The coverage dataset was used to create a heatmap (RStudio 2022.07.2, https://www.R-project.org, heatmap) against the reference genome RCC2590 to obtain a visual representation of coverage variations, after normalizing coverage by the read coverage of a standard invariant chromosome (chr 5). Windows where fewer than 10% of positions were covered by at least one read were categorized as deletions. Windows with single, 2-fold, or 3-fold coverage were delineated by merging all normalized coverage values across all strains and chromosomes. The 90% confidence interval for single-copy coverage was estimated using the qt function in RStudio (RStudio 2022.07.2), resulting in a range of 56% to 140% of the mean coverage. The same interval was applied to define 2-fold (140% to 224%) and 3-fold overage (224% to 308%) windows. Windows with more than 10% but <56% of covered positions were categorized as low coverage (Figs. 1, 2, S1, and S2).

### SNPs Analyses on the Callable Genome

Raw Illumina reads were quality checked with FastQC (FastQC_v0.11.7) and compared with MultiQC (MultiQC-1.14) (Andrews 2010; Ewels et al. 2016). The reads were trimmed with fastp (fastp-0.23.4) to remove adapters (–adapter_fasta) and N base (-n 0) and to conserve only reads longer than 80 bp (-l 80) (Chen et al. 2018; Chen 2023). The trimmed reads were mapped again, against RCC2590 with bwa-mem2 (version 2.2, mem) (Vasimuddin et al. 2019). The bam format was obtained using samtools (samtools-1.9, view, sort and index). Then, the duplicates were flagged using gatk MarkDuplicates (version 4.4.0.0) (Depristo et al. 2011). The SNPs were called with BCFtools (version 1.17) mpileup, call (−ploidy 1, because *Ostreococcus* are haploid) and filter (“QUAL>20 && DP > 100”). The vcf files were indexed with tabix. The callable genome was considered to be all the chromosomes except the SOC because of its repeat content. The DP4 parameter and the localization of the SNPs in the vcf files were used to select the SNPs above a certain frequency threshold. Since the sequencing came from a population of cells rather than a single cell, only SNPs with at 90% frequency were considered as fixed in a line. A binary matrix was created by generating a binary result (0: absence of the SNPs, 1: presence of the SNPs) by checking all the samples and the parent line's SNPs against the SNPs matrix. This matrix was then treated with RStudio (Rstudio 2022.07.2) using the Kimura (1980)) distance (hclust) to obtain the SNP phylogenetic dendrogram of Fig. 2. A matrix of all 111 SNPs and 9 short indels of the 11 samples identified was compiled and annotated with SNPeff (Cingolani et al. 2012) (Table S4). Additionally, to investigate difference in mutation rates between different chromosomal regions, we also estimated the number of SNPs segregating in each line, as sites with more than four reads confirming the alternative allele frequency (Table S1).

### Analysis of the “Accordion” Region of the SOC

Repeated regions were identified using a dotplot of the RCC2590 reference SOC sequence against itself (Geneious Prime 2023.0.4). The analysis was extended to all the stains SOC and the *O. tauri* RCC4221 SOC and confirmed by the long-read alignment. A blast of the RCC2590 pattern sequence against the RCC4221 pattern sequence was done to identify homologous sequences between the two species patterns. The main component of the pattern was identified as a long polymorphic sequence repeated in tandem using Geneious Prime 2023.0.4. The repeated sequence and the whole polymorphic tandem repeated region were blasted (blastn, tblastx, blastx) against the NCBI database (nt, nr/nt, internal transcripted spacer region) to identify homologous sequences. ORFinder (https://www.ncbi.nlm.nih.gov/orffinder/) was used to determine candidate ORFs in the whole polymorphic tandem repeated region. Candidate ORFs were classified depending on the position in the repeated sequence and the candidate amino-acid sequence. Each type of candidate ORF observed in all strains was aligned (global alignment with free-end gaps, cost matrix of 93% similarity, gap open penalty 12, gap extension penalty 3) and respective phylogenetic trees were built (Jukes-Cantor distance using neighbor-joining, iTOL 7.0 (Letunic and Bork 2024), as implemented in Geneious Prime 2023.0.4. The position and copy number of the spacer sequence were validated by long-read mapping against the assemblies. The protein structure of each type of candidate ORFs was determined by AlphaFold 3 (Abramson et al. 2024) and was compared with known or predicted structures using Foldseek (van Kempen et al. 2023). Domain and function were checked with InterProScan (Blum et al. 2025).

## Supplementary Material

evag162_Supplementary_Data

## Data Availability

The Genbank accession number of the O. mediterraneus reference genome is GCA_012295225.1, and the accession number of OmV2 MN688676. The accession number of the MA24 Illumina is SRX10812426. The accession numbers of the data generated and analyzed in this study are in project PRJEB90491, the ONT data accession numbers are from ERR15283078 to ERR1528387, and the Illumina data accession numbers: from ERR15283067 to ERR15283076.
