# Supplementary material for: Interchromosomal Translocations and Large Deletions Drive the Evolution of the Outlier Chromosome in the Smallest Photosynthetic Eukaryote
Source: Genome Biol Evol. 2026 Jul 2;18(7):evag162. doi: 10.1093/gbe/evag162 (PMC13390643; doi:10.1093/gbe/evag162)
Supplement: evag162_Supplementary_Data [file evag162_supplementary_data.zip › Fig-S1-S6.c.pdf]

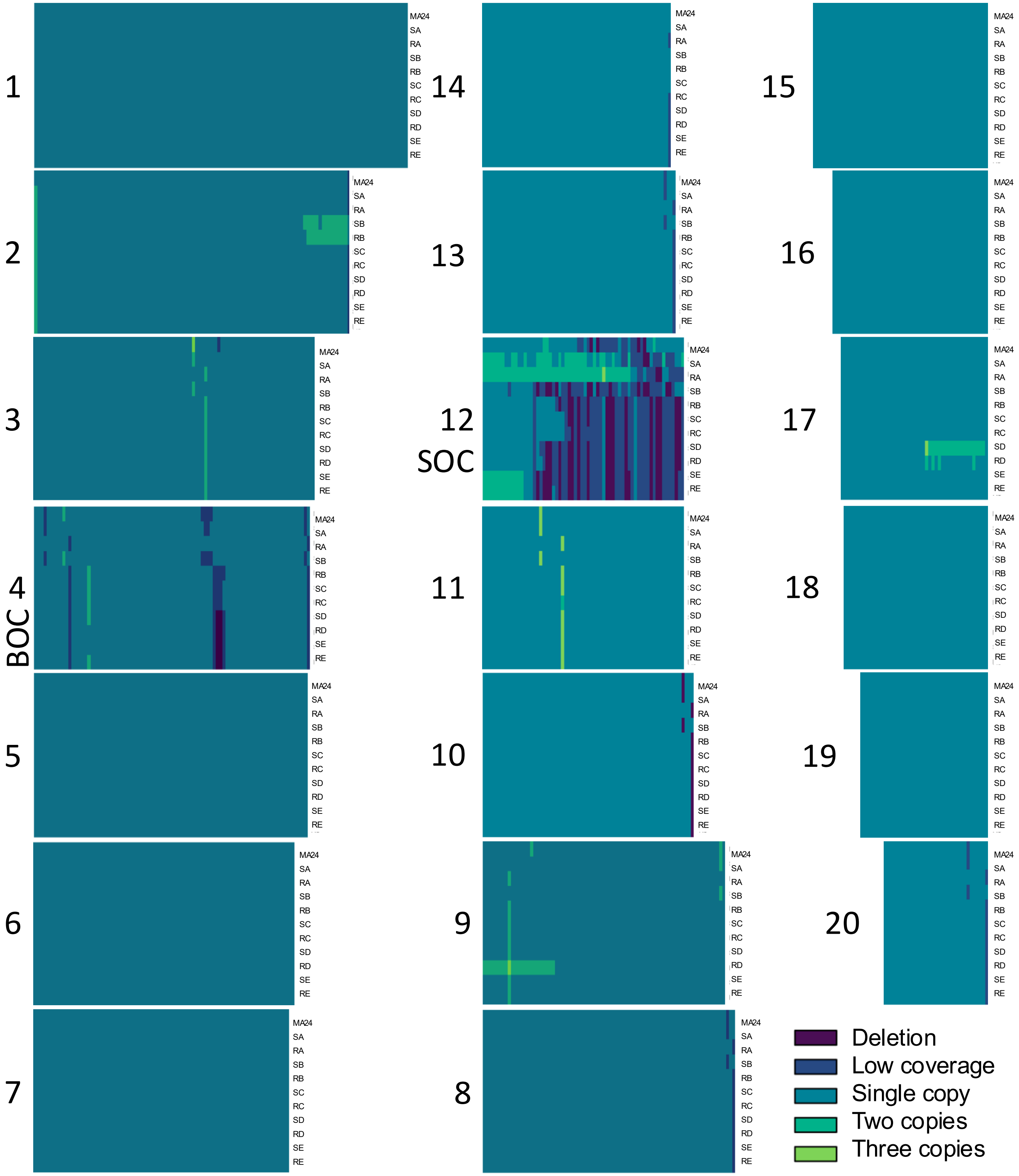

Figure S1: Detection of structural variations in 20 chromosomes of *O. mediterraneus*

Mapping of short reads for all 20 chromosomes of *O. mediterraneus* onto the reference *O. mediterraneus* RCC2590 genome for the parent MA24 and the five experimental-evolution lines analysed. Each line is designated as A–E, with virus-susceptible (S) and virus-resistant (R) pairs. The data from each experimental line were stacked for each chromosome. The coverage of each chromosome was normalised to the coverage of chromosome 5. Coverage was estimated over 10 kb

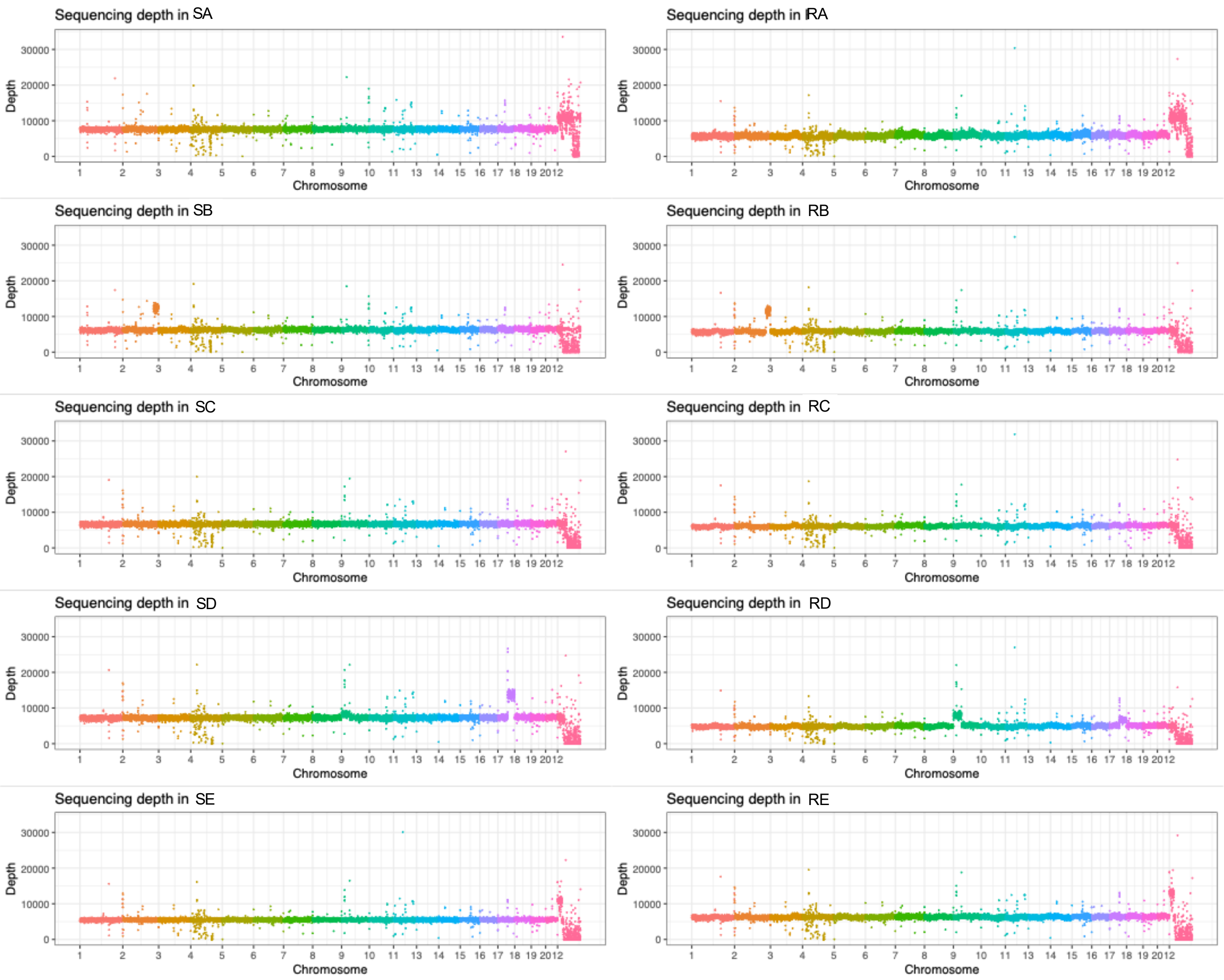

Figure S2: Coverage variation of the Illumina seq data from the 10 strains along 10kb windows on the 20 chromosomes of *O. mediterraneus RCC2590,* used for FigS1, Figure 1 and Figure 2.

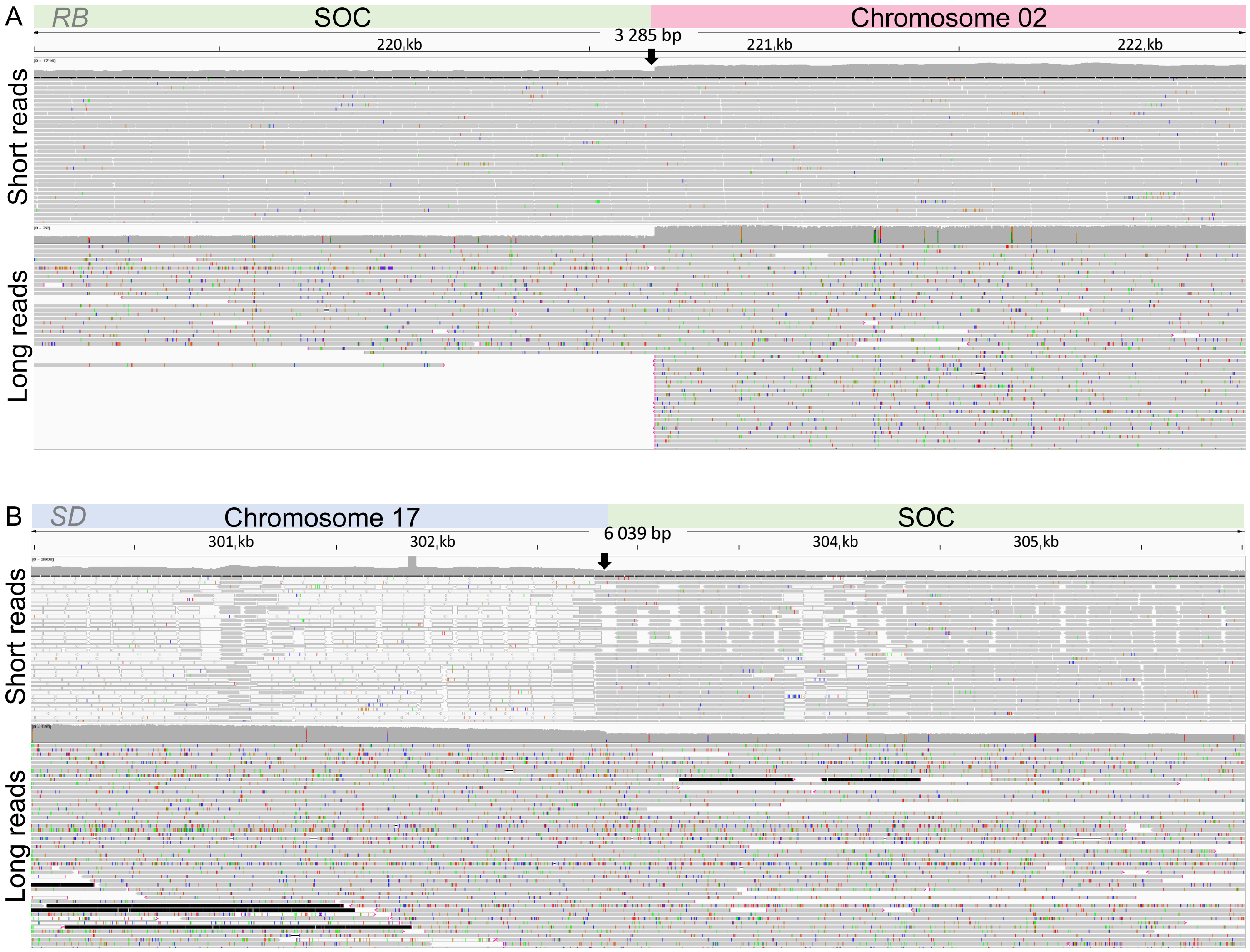

Figure S3: Short (bwa) and long read (minimap2) mapping onto the SOC-Ch02 fusion site of strain RB (A) and onto the SOC-C17 fusion site of strain SD (B), visualized using IGV (https://igv.org/).

A. RB – SOC assembly comparison between Masurca-Flye and Canu-Racon
(dottup word size 50 as implemented in Geneious 2026.0.2)

Masurca-Flye versus Canu-Racon SOC alignment.

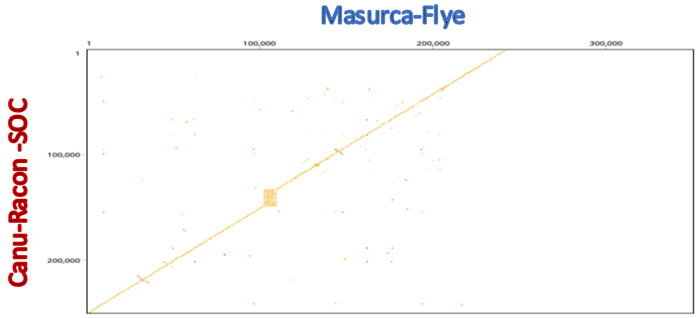

Canu-Racon versus reference Ch02 alignment

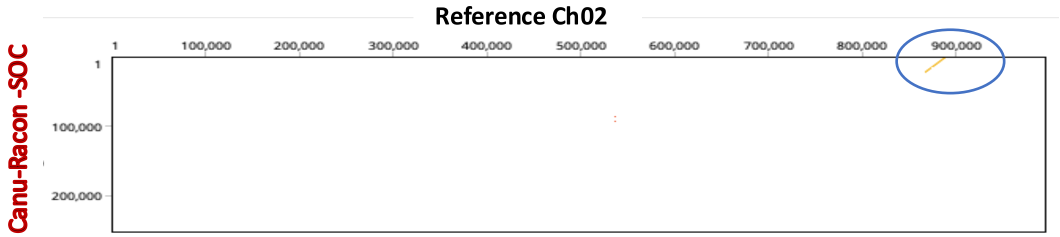

B. SD – SOC assembly comparison between Masurca-Flye and Canu-Racon
(dottup word size 50 as implemented in Geneious 2026.0.2)

Masurca-Flye versus Canu-Racon SOC alignment. The lack of alignment (blue circle) is due to the alignment to two different regions of ch17 after the fusion.

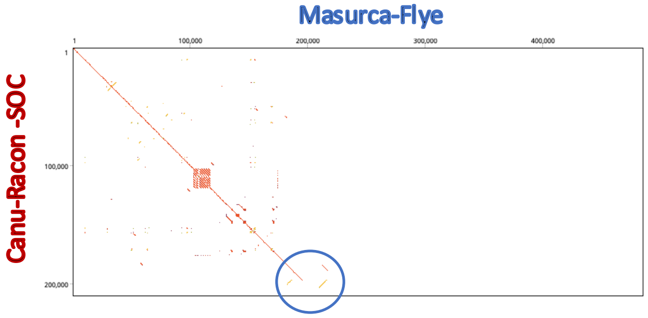

Canu-Racon versus reference Ch17 alignment

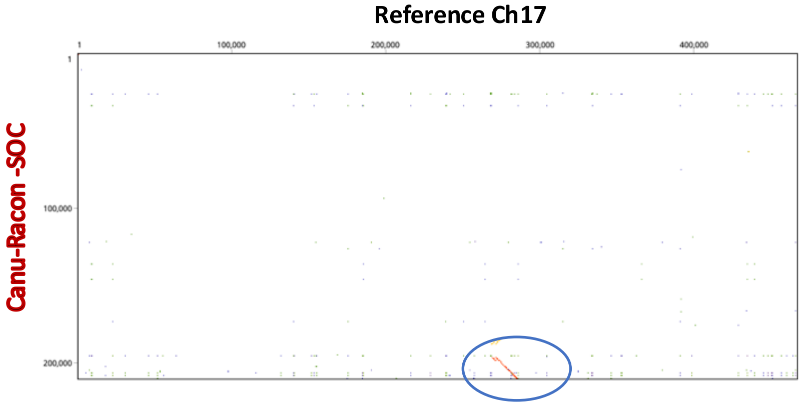

Fig S4. SOC assembly comparison between Masurca-Flye and Canu-Racon (A) strain RB (B) strainSD.

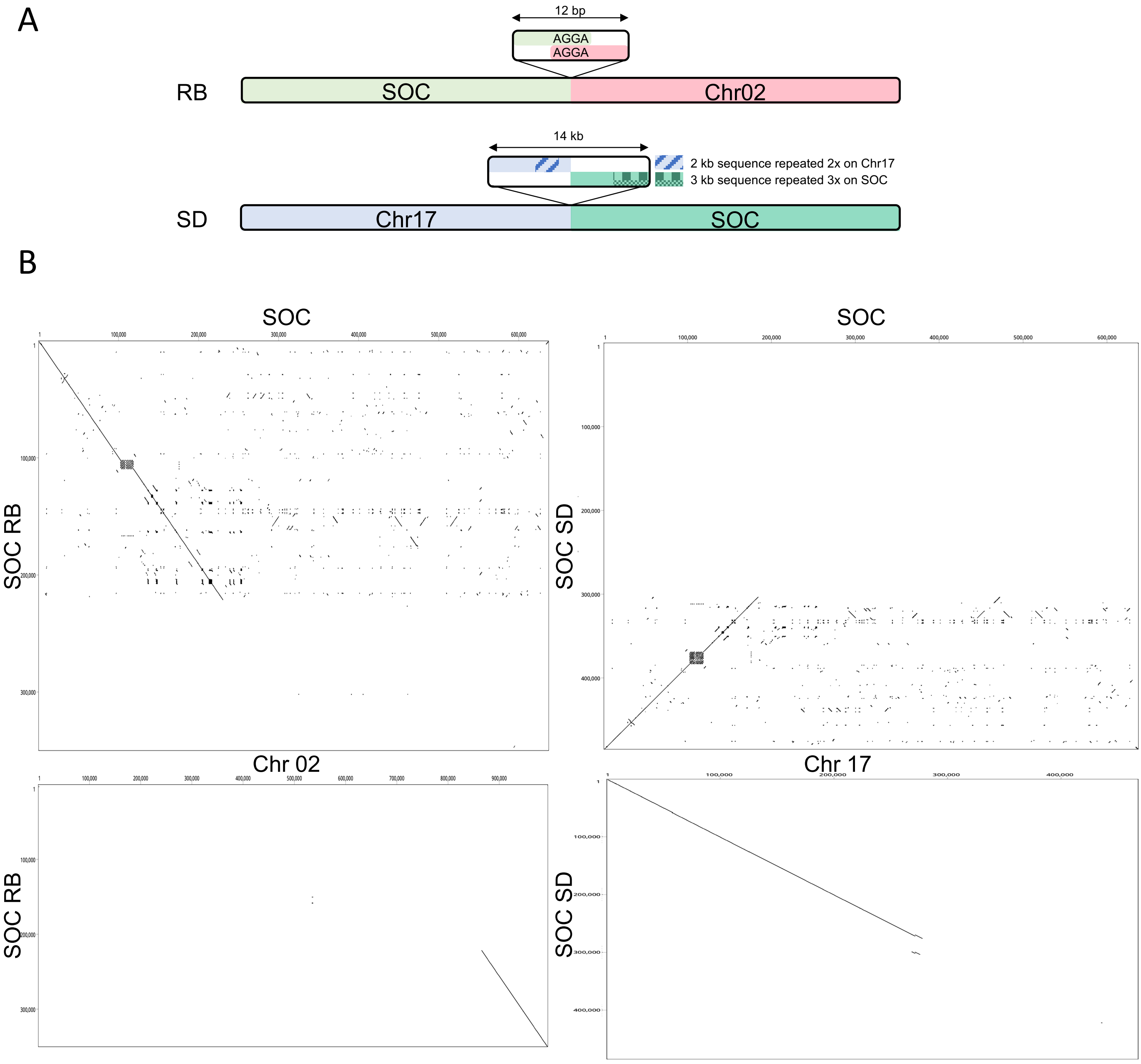

Fig S5. Zoom on the RB SOC-ch02 and SD SOC-ch17 junctions.

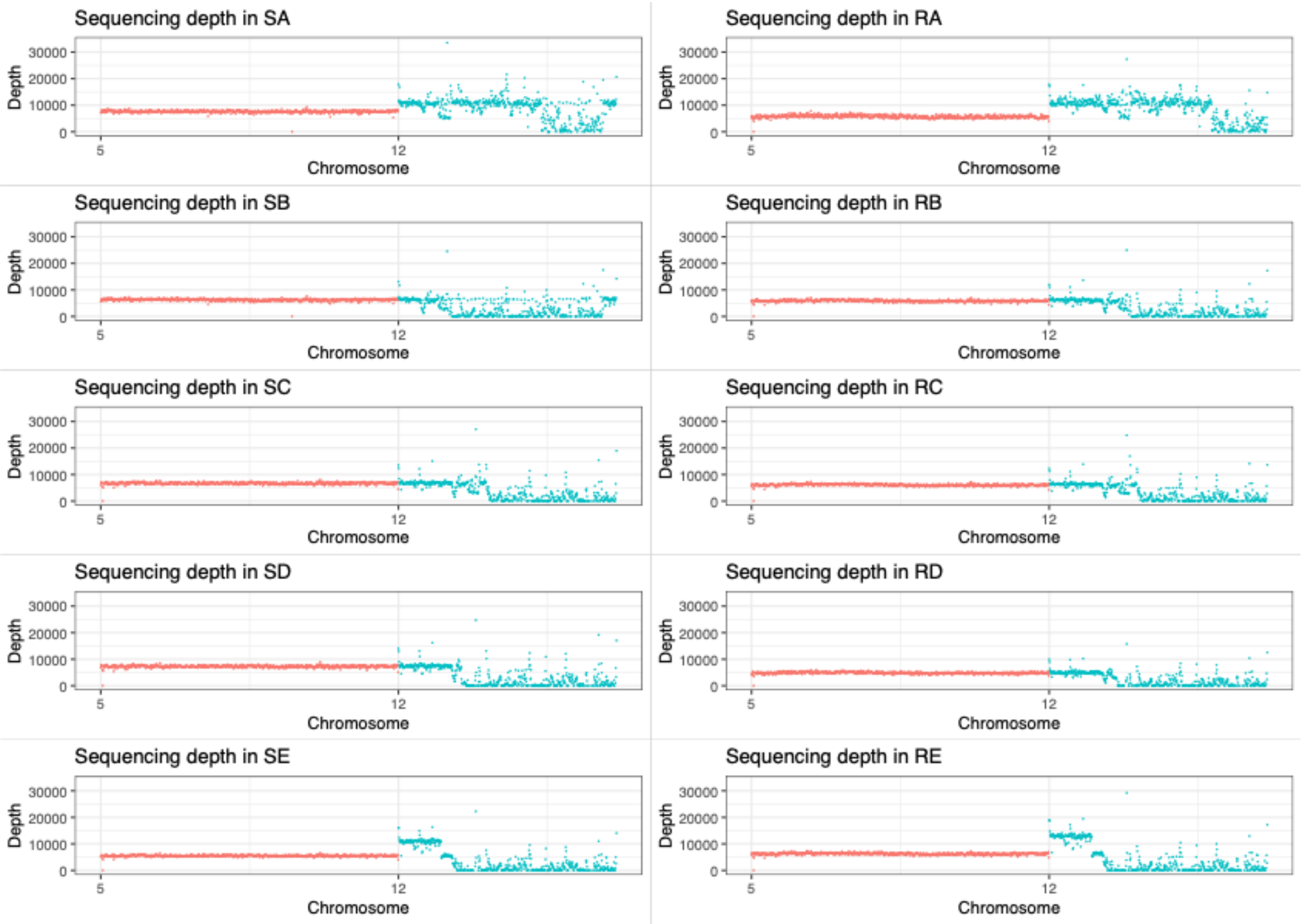

Figure S6: Raw read coverage variation along chromosome 5 (left, red) and chromosome 12 (right, blue) of *O. mediterraneus.* Each line is designated as A–E, with virus-susceptible (S) and virus-resistant (R) pairs.
